# The National Association of Dental Faculties

**Published:** 1888-07

**Authors:** 


THE NATIONAL ASSOCIATION OF DENTAL FACULTIES.

As the time for the annual meeting of the Association of Dental Faculties draws near, their prospective action becomes the subject of consideration. Rumors are rife of disaffection, and the possible withdrawal of some of the colleges from the Association. We have done our best to fathom these rumors, and find nothing positive in them. The general feeling among the colleges, so far as we have been able to make out, is that the organization deserves a still further trial. That misunderstandings have arisen, and that certain colleges have not lived up to the requirements of the Association, there can be no doubt, but these may in many instances be excused on the ground of a lack of a full understanding of the regulations adopted; all new organizations require time to adapt themselves to the needs for which they were intended. We should advise a spirit of leniency and charity for shortcomings at the present session. At the same time encourage full and free discussions in all cases. Petty jealousies should not be allowed to creep in and endanger the existence of an organization that gives promise of so much good in the future. Resolutions looking toward instructing the Association should be passed by the several societies that hold their sessions during the present month. The Illinois State Society led off in the right direction in the resolution offered by Dr. A. W. Harlan, and published in the June number of THE INDEPENDENT PRACTITIONER. Under our present system of education our colleges, to a large extent, are private institutions. The passage of laws looking to the control of the practice of dentistry have, in a measure, compelled the colleges to respect the statutes of the several States where laws have been passed. No State has, however, to our knowledge, taken any action toward establishing any set time which a student must fulfill in order to meet the requirements of the law. We must look to the colleges themselves for the adoption of a uniform course of instruction, which is so desirable, if we would have the degree represent any degree of uniformity.

A free expression upon the part of the laity, as represented in the dental societies of the country, would do much to bring about the desired end.

The journals of the country can also do much, and ought not to hesitate to publish any cases of bad faith on the part of any of the schools represented in this organization, should any such occur. The INDEPENDENT PRACTITIONER has borne its part in the past, and we will guarantee that its position in the future shall not be an equivocal one. We see in the Association of Dental Faculties an unlimited power for good. At the last meeting of the Association held at Washington, twenty-four colleges were represented. Such an organization of the corporate teaching bodies of our country has never before been known; and if it receives the hearty support of the profession, great things may be looked forward to from its action.

One of the most urgent demands at the present time is an extension of the course of study. Perhaps all that can be expected at the present is two full courses of seven months each; but all look forward to the time when three years of study shall be requisite to obtain a dental degree. Our students are rushed to death to fulfill the required curriculum now laid down in two short terms of four or five months each. Sufficient time is not allowed for the acquirement of practical laboratory training. Our students come to us now without the previous instruction formerly received in a preceptor's laboratory, and are immediately merged into large classes, where that personal instruction which is necessary to perfection cannot possibly be given in the short time required. It is true that students who have not had the advantage of previous study in private laboratories are required to attend a spring course of lectures, or else take a preceptor while attending their regular winter course. That the latter clause has been greatly abused is only too evident by looking over the catalogues of some of our most prominent schools. Professors are down as preceptors who have no laboratories, and in some instances are not even engaged in dental practice. We are fully convinced that a return to the former regulation of a year to be spent in a preceptor's laboratory would be beneficial. No institution can assume the same close relationship to the student that is possible by a practical dentist in general practice.

As it now stands, when a student comes to our institutions, he is turned over to the assistant demonstrators, who, as a rule, are undergraduates, or graduates of short standing. Such instruction cannot supplant personal instruction in private laboratories. The result of this change of base in our educational institutions has been decidedly detrimental, but few of the graduates of our colleges at the present day, who have depended upon the course of study prescribed therein, are competent metal-plate workmen. But few know anything practically about crown and bridge-work, and a less number can make a continuous gum or porcelain plate, unless they have taken additional special instruction from a specialist in that line and have paid an extra fee therefor. The majority of students graduate with the rudiments of dentistry only, and must look to private instruction for the completion of their dental education or go without. Under the existing conditions, which are only too palpable, how can any honest objection be made against the extension of the length of time required to complete the course of instruction? It has been argued that four or five months is a sufficient length of time for a lecturer on any dental subject to go over his branch thoroughly. If that is the case, the number of didactic lectures for each week should be decreased, and the student given more time for practical work in the laboratories. The present tendency of dental therapeusis is toward the antiphlogistic treatment of dental diseases. To understand correctly this line of treatment, it is absolutely essential that the student should have practical work in the histological and pathological laboratories—more attention should be given to the practical laboratory work in chemistry and dissection—more time for thorough preparation is urgently demanded for entrance upon the practice of one of the grandest specialties in medicine, but which can only be practiced as such by those who have earned the right to such a position by broad culture.

